# Providing a supplemental source of water or a trace-mineral-based drinking solution upon feedlot arrival affects intake, growth performance, and health of newly received finishing calves

**DOI:** 10.1093/jas/skag022

**Published:** 2026-02-02

**Authors:** Mackenzie M Smithyman, Vinícius N Gouvêa, Dayna L Campbell, Glenn C Duff, Clint A Löest, Reinaldo F Cooke, Matthew R Beck, Mark E Branine

**Affiliations:** Department of Animal and Range Sciences, Clayton Livestock Research Center, New Mexico State University, 15 NMSU Ln, Clayton, NM 88415, United States; Texas A&M AgriLife Research, 3211 Russell Long Blvd, Canyon, TX 79015, United States; Department of Animal Science, Texas A&M University, 474 Olsen Blvd Suite 133, College Station, TX 77843, United States; Department of Animal and Range Sciences, Clayton Livestock Research Center, New Mexico State University, 15 NMSU Ln, Clayton, NM 88415, United States; Department of Animal and Range Sciences, Clayton Livestock Research Center, New Mexico State University, 15 NMSU Ln, Clayton, NM 88415, United States; Department of Animal and Range Sciences, New Mexico State University, 2980 S. Espina St, Knox Hall, Room 202, Las Cruces, NM 88003, United States; Department of Animal Science, Texas A&M University, 474 Olsen Blvd Suite 133, College Station, TX 77843, United States; Department of Animal Science, Texas A&M University, 474 Olsen Blvd Suite 133, College Station, TX 77843, United States; Zinpro Corporation, 7500 Flying Cloud Dr, Eden Prairie, MN 55344, United States

**Keywords:** beef cattle, bovine respiratory disease, hydration, immune response, inflammation, stress

## Abstract

The objective of this study was to evaluate the effects of providing a supplemental water source (SWS) or an experimental nutrient repletion solution (NRS) following feedlot arrival on intake, growth performance, health, and immune responses of newly received calves. A total of 270 weaned lightweight British × Continental crossbred heifers (initial body weight [BW] = 236 ± 19 kg) were ranked by shrunk BW and allocated into 18 soil-surfaced pens (12 × 35 m; 15 heifers/pen). Treatments were: 1) Control (CON): water was provided through a standard in-pen automatic waterer only; 2) Supplemental water source (SWS): CON plus water provided with one additional 416-L stock tank/pen; 3) Experimental nutrient repletion solution (NRS): provided with one 416-L stock tank/pen as the only source of drinking solution. The SWS and NRS were provided from days 1 to 4, after which supplemental tanks were removed. From days 5 to 56, all heifers had only access to the standard in-pen automatic waterer. Heifers had *ad libitum* access to feed and water and the WI was measured daily throughout the experiment. Body weights and blood samples via jugular venipuncture were collected on days 1, 4, 14, 28, and 56. A treatment × day interaction was observed for average BW, ADG, and water intake (*P *≤ 0.05). SWS and NRS increased DMI compared to CON during days 5–15 (*P *< 0.001), and the increase in DMI persisted for SWS compared to CON between days 16 and 29 (*P *< 0.01). The ADG was lower for NRS compared to CON during days 1 to 4 (*P *< 0.01), and water intake was greater for SWS and NRS compared to CON between days 1 and 4 (*P *< 0.001). No differences between treatments were observed for morbidity and mortality rate (*P *≥ 0.28). The cumulative incidence of BRD tended to be lower for SWS compared to CON during days 9 to 27 (*P *≤ 0.10). No treatment or treatment × day interactions were observed for any of the plasma hormones and metabolites evaluated (*P *≥ 0.11), except for plasma glucose, which tended to be lower for NRS compared to CON (*P *= 0.10), and serum antibody titers against bovine viral diarrhea virus, which were greater for NRS compared to CON (*P *= 0.02). Providing SWS or NRS to high-risk newly received beef calves for 4 d after arrival to the feedyard may increase water, feed intake and immune response.

## Teaser

Newly received feedlot calves may benefit from additional access to in-pen water sources after feedlot arrival.

## Introduction

Bovine respiratory disease (BRD) is the most diagnosed and economically costly detrimental disease affecting the US beef cattle industry (Galyean et al. 2022). The BRD complex is a multifaceted disease brought on by a combination of bacterial and viral pathogens resulting from weaning and receiving stressors (Galyean et al. 2022). The receiving or starting period is defined as the first 4 to 8 wk following arrival during which time beef calves are transitioned to the feedlot environment, particularly to the novel changes in nutritional management (Richeson et al. 2019). According to Smith (1998), the first 45 d after feedlot entry account for 65% to 80% of all morbidity caused by respiratory illness, costing the US beef cattle industry an estimated $900 million annually (Peel 2020). Newly received feedlot cattle are frequently subjected to physiological and psychological stressors associated with prolonged periods of restricted access to feed and water due to marketing and transportation, resulting in decreased performance and health (Cooke 2017). Stressors as well as prolonged periods of deprivation to feed and per or water associated with receiving processes such as marketing and transportation can cause a decrease in dry matter intake (DMI) and water intake, which can exacerbate the adverse effects of stress on immune function, ­decrease performance, and put cattle at greater risk for BRD (Duff and Galyean 2007; Galyean et al. 2022).

Low nutrient intake and dehydration are often accompanied by depletion of several nutrients with water being the most critical (Wagner and Engle 2021). Additional losses of electrolytes and trace elements may compromise immune function and make the animal more susceptible to respiratory pathogens (Galyean et al. 1999; Clark et al. 2006). Likewise, deprivation of feed and decreased appetite may also reduce the animal’s overall energy balance (Galyean et al. 1999). Supplementing a readily available energy source along with minerals and other essential nutrients in a readily available water-soluble solution can improve overall nutrient repletion and potentially reduce the recovery time of nutrients lost during transport when compared to deionized water (Cole 1996). Carey et al. (2017) concluded that providing an energy source such as glycerin through drinking water can influence hydration status and metabolic responses in stressed cattle, highlighting the potential of water-based supplementation strategies to support nutrient replenishment and immune function in newly received calves. The use of oral hydration therapy in high-risk receiving calves upon feedlot arrival has been reported to improve performance and health of newly received feedlot calves (Lopez et al. 2018; Tomczak et al. 2019). However, little is known regarding providing a nutrient repletion solution through the water upon feedlot arrival and how it may influence hydration status, performance, and morbidity of calves during the receiving period.

We hypothesized that increasing access to water or providing an experimental nutrient repletion solution (NRS) upon feedlot arrival would improve the hydration status of newly received calves and help replete nutrients, which could increase feed intake and decrease morbidity and mortality from BRD. This study aimed to evaluate the effects of providing a supplemental water source or an NRS following feedlot arrival on intake, growth performance, health, and immune responses of newly received calves.

## Materials and methods

This experiment was conducted at the New Mexico State University, Clayton Livestock Research Center, Clayton, New Mexico. All procedures involving the care and use of animals were approved by the New Mexico State University Institutional Animal Care and Use Committee (protocol #2020-028).

### Animals and facilities

A total of 270 weaned lightweight British × Continental crossbred heifers were sourced from southeast commercial auctions and transported approximately 1,100 km, averaging 11 h on the truck to the Clayton Livestock Research Center in Clayton, NM. Heifers were shipped in 2 truckloads with 90 heifers per truckload in February 2021 (Run 1; *n* = 180; initial body weight [BW] = 237 ± 23 kg), and one truckload with 90 heifers in August 2021 (Run 2; *n* = 90; initial BW = 234 ± 15 kg). Except for the diet composition used in each run, all procedures and methods were the same for both runs. Environmental conditions, primarily temperature, precipitation, wind speed, and humidity, were recorded daily for both runs from the Clayton Municipal Airpark weather station (Table 1), located approximately 10 km from the Clayton Livestock Research Center.

**Table 1:** Mean monthly environmental variables observed during the 56-d receiving period for each run^1^.

	Run 1^2^			Run 2^3^		
Item	February	March	April	August	September	October
**Temperature, °C**						
**Average**	2.50	9.70	10.1	22.5	21.9	16.7
**Maximum**	16.7	17.2	17.8	30.0	37.2	30.0
**Minimum**	−7.80	1.70	2.20	15.0	6.10	5.00
**Total precipitation, mm**	0.00	69.6	25.1	1.00	31.0	61.2
**Average wind speed, km/h**	23.5	26.1	33.0	26.7	25.7	19.6
**Average humidity, %**	25.2	46.0	32.7	38.5	35.4	54.9
**Temperature-humidity index^4^**	45.3	52.0	53.1	67.6	66.7	61.1

1 Temperature, precipitation, wind speed, and humidity, were recorded daily for both runs from the Clayton Municipal Airpark weather station, located approximately 10 km from the Clayton Livestock Research Center.

2 February to April 2021 (Run 1; *n* = 180 heifers; 15 heifers/pen; 12 pens total and 4 pens/treatment).

3 August to October 2021 (Run 2; *n* = 90 heifers; 15 heifers/pen; 6 pens total; 2 pens/treatment).

4 According to Perdomo et al. (2020). Temperature-humidity index (THI) = [(1.8 × ambient temperature) + 32]—[0.55—(0.0055 × relative humidity)] × [(1.8 × ambient temperature)—26].

Heifers were shrunk for an average of 11 hours during transportation. Upon feedlot arrival, all heifers were processed before being given access to feed and water. A Bud Box System with a double alley (Daniels Manufacturing Co., Ainsworth, NE) leading to a hydraulic squeeze chute (Ultimate AH-10; Daniels Manufacturing Co.) mounted on load cells (Avery Weigh-Tronix, Fairmount, MN) was used to restrain and collect individual BW throughout the study. On day 1, heifers were weighed and vaccinated against respiratory disease (2.0 mL s.c. injection of Vista Once SQ, Merck Animal Health, Millsboro, DE) and clostridiosis (5.0 mL s.c. injection of Covexin 8, Merck Animal Health), for internal parasites administered both an oral dewormer (2.5 mL/50 kg of BW, Safe-Guard, Merck Animal Health) and injectable dewormer (7.0 mL s.c. injection of Dectomax; Zoetis, Parsippany, NJ). Heifers received 200 mg testosterone propionate and 20 mg estradiol benzoate implant (Synovex H, Zoetis) and given a unique identification number. Heifers were then ranked by shrunk BW and hair coat color, given a pen tag, and allocated into one of 18 soil-surfaced pens (12 × 35 m; 15 heifers/pen) with 11 m (approximately 73 cm linear bunk space/heifer) of bunk space.

### Experimental design and treatments

The experiment was a randomized complete block design, with pens of cattle serving as the experimental unit. The experiment consisted of 3 blocks (corresponding to the 3 truckloads; 2 truckloads from run 1 and 1 truckload from run 1) and 3 experimental treatments, with 2 replicated pens per treatment within each block. Therefore, there were 18 pens, with a total of 6 pens per treatment. Within each block, heifers were ranked by initial BW and hair coat color and allocated into 1 of 6 pens to ensure that all pens had equivalent shrunk BW, and then treatments were randomly assigned to pens.

Experimental treatments were: Control (CON): water was provided through a standard in-pen automatic waterer only (CattleMaster 480; Richie Inc., Conrad, IA; 8.6 cm water trough space per head); Supplemental water source (SWS): CON plus water provided with one additional 416-L stock tank/pen (KMT 100; Tuff Stuff, Terra Bella, CA; additional 9.0 cm water trough space per head); Experimental nutrient rehydration solution (NRS): provided with one 416-L stock tank/pen (KMT 100; Tuff Stuff; 9.0 cm water trough space per head) as the only source of drinking solution (Table 2). The SWS and NRS were provided from days 1 to 4, after which supplemental tanks were removed. From days 5 to 56, all heifers had only access to a standard in-pen automatic waterer (CattleMaster 480; Richie Inc.).

**Table 2:** Chemical composition (mg/L) of water and the novel repletion solution (NRS) supplied to heifers for 4-d following feedlot arrival.

Item	Water^1^	NRS^1^,^2^
**S**	13.0	18.2
**Na**	21.0	111
**Ca**	43.3	50.5
**Mg**	27.5	124
**K**	5.00	366
**Fe**	<0.05	0.77
**Mn**	<0.005	2.13
**CaCO_3_**	220	640
**B**	0.10	0.10
**Cu**	<0.01	1.94
**Mo**	<0.01	<0.02
**Zn**	0.05	10.7
**Acetylsalicylic acid^3^**	-	700
**Glycerol^3^**	-	9,500
**Propylene glycol^3^**	-	10,000

1 Chemical analysis was performed by Servi-Tech Laboratories, Amarillo, TX. One composite sample from days 1 to 4 was analyzed.

2 Treatments were Control (CON): water was provided through standard in-pen automatic waterer only (CattleMaster 480; Richie Inc., Conrad, IA); Supplemental water source (SWS): CON plus water provided with one additional 416-L stock tank/pen (KMT 100; Tuff Stuff, Terra Bella, CA); Nutrient repletion solution (NRS): provided with one stock tank/pen (KMT 100; Tuff Stuff) as the only source of drinking solution. Treatments were provided from days 1 to 4, after which supplemental tanks were removed. From days 5 to 56, all heifers had only access to the standard in-pen automatic waterer (CattleMaster 480; Richie Inc.).

3 Based on the inclusion rate.

### Feed intake management

Once processed, heifers were distributed into pens and had *ad libitum* access to feed and water throughout the experiment. For Run 1, heifers were fed a complete commercial starter ration (RAMP; Cargill Sweet Bran, Dalhart, TX; Table 3), and for Run 2 heifers were fed a standard receiving diet containing 30% roughage and 70% concentrate (DM basis; Table 4). In both runs, heifers were fed the receiving diets once daily at 0700 h, and bunks were evaluated at 0630 h for unconsumed, residual feed. Feed delivery was adjusted throughout the study based on morning bunk evaluation so that feed bunks contained trace amounts of residual feed. Refusals were removed daily at 0630 h on days 1 to 4, weighed, and a sample was collected for dry matter (DM) determination and DMI calculations. After day 4, feed bunks were managed so that roughly 3% feed refusals from the previous day remained in the bunk, and the feed call was adjusted based on the visual amount of unconsumed feed. When necessary, refusals were removed, and weighed, and samples were collected for DM analyses and DMI calculation.

**Table 3:** Nutrient composition (dry matter basis) of the complete starter fed to heifers during a 56-d feedlot receiving period (Run 1)^1^,^2^.

Item	Composition, %
**Dry matter, % as fed**	68.1
**Crude protein, %**	21.8
**Acid detergent fiber, %**	19.6
**Neutral detergent fiber, %**	38.9
**Ash, %**	9.60
**Ca, %**	1.46
**P, %**	0.79
**Mg, %**	0.41
**K, %**	1.55
**Na, %**	0.20
**Zn, mg/kg**	131
**Fe, mg/kg**	355
**Mn, mg/kg**	67.2
**Cu, mg/kg**	23.2

1 Chemical analysis was performed by Cumberland Valley Analytical Services, Inc., Waynesboro, PA (*n* = 4).

2 RAMP (Cargill Corn Milling, Dalhart, TX) is a proprietary starter feed that contains a high level of wet corn gluten feed and a minimal amount of forage (Schneider et al. 2017).

**Table 4:** Ingredient and chemical composition (dry matter basis) of the experimental diet fed to heifers during a 56-d feedlot receiving period (Run 2)^1^.

Item	Inclusion, %
**Ingredient**	
**Steam-flaked corn**	35.9
**Wheat hay**	30.0
**Wet corn gluten feed^2^**	17.0
**Dried distillers’ grain**	15.0
**Limestone**	1.60
**Salt**	0.33
**Trace mineral and vitamin premix^3^**	0.22
**Analyzed nutrient composition**	
**Dry matter, % as fed**	81.0
**Crude protein, %**	17.0
**Acid detergent fiber, %**	15.1
**Neutral detergent fiber, %**	29.1
**Ash, %**	8.90
**Total digestible nutrients, %^4^**	80.2
**Net energy of maintenance, Mcal/kg^4^**	1.94
**Net energy of gain, Mcal/kg^4^**	1.23
**Ca, %**	0.88
**P, %**	0.55
**Mg, %**	0.25
**K, %**	1.21
**Na, %**	0.24
**Zn, mg/kg**	103
**Fe, mg/kg**	332
**Mn, mg/kg**	74.7
**Cu, mg/kg**	16.3

1 Chemical analysis was performed by Cumberland Valley Analytical Services, Inc., Waynesboro, PA (*n* = 4).

2 Sweet Bran (Cargill Corn Milling, Dalhart, TX).

3 Trace mineral and vitamin premix containing (dry matter basis): 70 mg/kg Co, 4,620 mg/kg Cu, 255 mg/kg I, 11,000 mg/kg Mg, 70 mg/kg Se, 21,000 mg/kg Zn, 1,000,000 UI/kg Vit. A, 130,000 IU/kg Vit. D, 18,000 IU/kg Vit. E.

4 Calculated using tabular values from NASEM (2016).

The diets were delivered using a horizontal rotary feed wagon (Roto-Mix model 274 12B; Roto-Mix, Dodge City, KS) containing a digital scale (Scale-Tec Point; Scale-Tec, Anamosa, IA), and hauled by a tractor (John Deere model 6715; John Deere, Moline, IL). The commercial starter ration (RAMP) used in Run 1 was delivered at the research site every 15-d, ready to use. Wheat hay, steam-flaked corn, and wet corn gluten feed used in Run 2 were loaded directly into the feed wagon. Dried distillers’ grains plus solubles, limestone, salt, trace mineral and vitamins premix, were individually weighed into plastic buckets using a digital platform scale with 0.05 kg resolution (Angel SS-400, Angel POS, Burnaby, Canada) and then loaded into the feed wagon that mixed the feed thoroughly for 5 minutes prior to being delivered to each pen. Due to its effect on DMI (National Academies of Sciences, Engineering, and Medicine (U.S.). Committee on Nutrient Requirements of Beef Cattle 2016), monensin was not included in the diets used in the current experiment.

### Water intake management

Water intake from the in-pen automatic waterers was measured 3 times daily at 0700 h, 1200 h, and 1900 h throughout the trial using a digital flow meter (Fill-Rite FR1118P10; Fort Wayne, IN) attached to the waterers. The supplemental 416-L stock tanks were calibrated prior to the study initiation to determine the linear relationship of tank depth and tank volume, completed by measuring the depth of water in the tank at 5 points following the addition of a known quantity of water. The linear regression coefficient was then established, allowing the amount of water remaining in the tanks to be determined by measuring the depth in cm:


(1) Water volume (L)=8.52945 × average depth (cm)

Root mean square error (root MSE) was 4.46, the adjusted R-square was 0.998, and the coefficient of variation was 2.39. The stock tanks were then measured manually at 5 points using a stainless-steel long-length ruler (120 cm) and water intake (L/head) from each tank was calculated as:


(2) $$\mathrm{Water}\;\mathrm{Intake}\;(L)=\frac{\mathrm{Initial}\;\mathrm{volume}\;(L)\;-\;\mathrm{final}\;\mathrm{volume}\;(L)}{\mathrm{head}}\mathrm{count}$$

Before the start of each run, all water sources were evaluated for potential water loss due to evaporation. After a 12 h evaporation period evaluation, the amount of water lost because of environmental factors was not detectable and assumed to be negligible in this study. Supplemental stock tanks were replenished 3 times daily using two 1,200 L horizontal leg tanks (Norwesco Inc., St. Bonifacius, MN) attached with a portable transfer utility pump (PDS-30, Exreaup). A concentrated solution containing the NRS treatment, with organic source of trace minerals (Zinpro Corporation, Eden Prairie, MN), was prepared daily from day 1 to day 4. Each ingredient was weighed using a bench scale with 0.05 kg resolution (Ohaus 30031708, Ohaus Corporation, Parsippany, NJ) into a 1 L beaker, mixed using a magnetic stirrer, and then added to respective treatment tanks containing 370 L of water, to maintain proper concentration in drinking solution during treatment days.

### Sample collections and health evaluations

Unshrunk BW was collected on days 4, 14, and 28. Shrunk BW was collected on days 1 and 56 after 16 hours of feed and water withdrawal. A cohort of 5 heifers from the average initial BW in each pen was selected on day 1 for blood collection via jugular venipuncture on days 1, 4, 14, 28, and 56. Serum samples were collected using 8.5 mL vacutainers (BD Vacutainer model 368016, Becton, Dickinson and Co., Franklin Lakes, NJ) and allowed to clot at room temperature (20 to 25 °C) for 60 minutes. To prevent clotting and metabolic changes caused by blood cell enzymes, samples intended for plasma were collected in 10 mL vacutainers containing lithium heparin (BD Vacutainer #367880) and kept on ice. Trace element plasma samples were collected in 6 mL vacutainers (BD Vacutainer #36838) containing K_2_ EDTA. Serum and plasma tubes were then centrifuged (2,500 × *g* at 4 °C) for 35 min and all samples were then aspirated and placed into their respective 2 mL microtubes and frozen at −80 °C until analyzed. Plasma samples were also collected in 3 mL vacutainers (BD Vacutainer #367856) containing K_2_ EDTA for complete blood counts.

Two trained evaluators monitored animal health daily throughout the study by implementing a 4-point scale method based on depression, anorexia, respiratory, and temperature (“DART”) as described by Step et al. (2008) and Wilson et al. (2015). Briefly, DART score 0 is a healthy calf, while a DART score 1 is a calf slow moving, but not necessarily depressed. DART score 2 is a calf with possible nasal or ocular discharge, and depressed. DART score 3 is a calf that is visibly depressed, has nasal and or ocular discharge, a lowered head, and is isolated. A calf with a DART score of 4 has limited mobility, is depressed, noticeably thin, and has nasal and or ocular discharge. Heifers observed with signs of morbidity were briefly removed from their pens and restrained in the chute to further assess if antimicrobial treatment was necessary. Heifers with a severity score of 1 or 2 and rectal temperature of 40 °C or greater received medical treatment. No medical treatment was provided if heifers with a severity score of 1 or 2 had a rectal temperature below 40 °C. However, heifers with a severity score of 3 or 4 were provided medical treatment regardless of rectal temperature above or below 40 °C. Heifers receiving their initial medical treatment were given florfenicol antibiotic with flunixin meglumine (Resflor Gold, Merck Animal Health, Madison, NJ). If a second treatment was warranted, heifers received an injection of ceftiofur crystalline-free acid (Exceed, Zoetis, Parsippany, NJ). If a third treatment was justified, heifers were removed from the study, placed in the hospital pen, and given an injection of oxytetracycline (Bio-Mycin 200, Boehringer Ingelheim, Inc., St. Joseph, MO). Heifers administered antibiotics were dosed in accordance with the directions on the label with all relevant withdrawal times observed. All heifers were on a 5-d moratorium after each treatment before qualifying for subsequent antibiotic treatment. After each medical treatment, heifers received an ear tag to designate the last medical treatment.

### Laboratory analysis

The complete blood count was determined within 60-min after blood collection using an automated veterinary 5-part differential hematology analyzer (VetScan HM5; Abaxis, Zoetis, Parsippany, NJ). A blank measurement and quality control were used to verify the analyzer’s cleanliness and accuracy, respectively, before each sampling day.

Plasma samples from days 1, 4, 14, 28, and 56 were analyzed for concentrations of glucose using a reagent set kit (Ref. G7521-500, Point Scientific; Canton, MI) and optical density was read at 500 nm using a multi-mode plate reader (Synergy LX, BioTek; Winooski, VT). Plasma samples from sample days 1, 4, and 14 were analyzed for concentrations of bovine non-esterified fatty acids (NEFA) using a commercial enzyme-linked immunosorbent assay (ELISA) kit (Cat. No. MBS748204, My BioSource; San Diego, CA) and optical density was read at 450 nm using a microplate reader (Synergy LX).

Serum samples from days 1, 4, and 14 were analyzed for concentrations of bovine TNF-alpha using a commercial ELISA kit (Cat. No. CSB-E12020B, Cusabio; Houston, TX), and optical density was read at 450 nm using a microplate reader (Synergy LX).

All plasma samples were analyzed for concentrations of cortisol using radioimmunoassay (RIA) kit (#07221106, MP Biomedicals, Santa Ana, CA; Colombo et al. 2019), haptoglobin using a commercial ELISA kit (#2410-7; Life Diagnostics, West Chester, PA; Cooke and Arthington 2013) and optical density was read at 450 nm using a microplate reader (Synergy LX). The standard curve was constructed by plotting the absorbance values of the standards vs. the log of the concentrations. All samples were run in duplicate.

All serum samples were analyzed for β-hydroxybutyrate (BHB), lactate, ɣ-glutamyl transferase (GGT), urea nitrogen (BUN), and bicarbonate using an automated blood chemistry analyzer (Carysta High Volume Chemistry Analyzer; Zoetis, Parsippany, NJ). Samples were analyzed in a single assay with an intra-assay CV of < 5%.

The intra-assay and inter-assay CV were 6.1% and 6.6% for NEFA, 5.3% and 6.5% for TNF-alpha, 0.8% and 3.7% for glucose, 4.2% and 4.4% for cortisol, 4.4% and 8.6% for haptoglobin, 8.7% and 4.3% for parainfluenza-3 virus, 5.1% and 3.4% for bovine respiratory syncytial virus, and 6.6% and 5.6% for bovine viral diarrhea viruses, respectively.

Plasma trace mineral concentration on days 1, 4, 14, 28, and 56 were analyzed by Zinpro Corporation (Eden Paririe, MN). The Zinpro standard operating procedure for quantitating total mineral content in plasma samples is intended for research and informational purposes by Zinpro or its affiliates. Briefly, plasma samples were digested in concentrated acid (HNO_3_ and HCl) and then diluted with high-performance liquid chromatography grade H_2_O to the necessary volume. Calibration standards and quality control (QC) samples were generated in a dilute nitric acid matrix. Standards and samples were analyzed using inductively coupled plasma mass spectrometry with MassHunter quantitative analysis software (7800 ICP-MS, Agilent; Santa Clara, CA). The calibration range varied depending on the analyte, experimental expectations, and dilution parameters, but it was typically between 1 ppb and 1 ppm. Lower or higher standards were used depending on the needs of the analysis. Internal standards were used to indicate potential matrix interferences. Internal standard recovery for samples (particularly unknown samples) were 80% and 120% of calibration standards. Quality control samples of known concentration were taken every ten samples to ensure that the assay calibration was maintained. The recovery rate of QC samples was between 90% and 110% of the target value.

Samples of diets and feed ingredients were collected every 2 wk throughout the duration of the feeding period. At the end of the experiment, samples were thawed, pre-dried at 55 °C for 72 hours using a forced-air drying oven and grounded to pass through a 1-mm screen using a Wiley mill (Thomas Scientific, Swedesboro, NJ) and then analyzed for DM (method 930.15; Association of Official Analytical Chemists (AOAC) 2000), crude protein (CP; method 990.03; Association of Official Analytical Chemists (AOAC) 2000) using a nitrogen combustion analyzer (FP828, Leco Corporation, St. Joseph, MI), neutral detergent fiber with sodium sulfite and α-amylase (NDF; Van Soest et al. 1991, modified to use Whatman 934-AH glass micro-fiber filters with 1.5 µm particle retention), acid detergent fiber (ADF; method 793.18; Association of Official Analytical Chemists (AOAC) 2000), minerals (Ca, P, Mg, K, Na, Zn, Fe, Mn, Cu; method 985.01; Association of Official Analytical Chemists (AOAC) 2005), and ash (method 942.05; Association of Official Analytical Chemists (AOAC) 2000) at a commercial laboratory (Cumberland Valley Analytical Services; Waynesboro, PA; Tables 3 and 4). In addition to the feed analysis, samples of water and NRS solution were collected daily from days 1 to 4 and a composite sample of each treatment was submitted to a commercial laboratory (ServiTech Laboratories, Amarillo, TX) for evaluation of mineral content using Inductively Coupled Plasma-Atomic Emission Spectroscopy analysis (Table 2).

### Statistical analysis

All data were analyzed as a randomized complete block design with pen as the experimental unit for all the analyzed variables. Quantitative data were analyzed using the MIXED procedure of SAS (SAS Institute Inc., Cary, NC) whereas binary data were analyzed using the GLIMMIX procedure (SAS Institute Inc.) with a binomial distribution and logit link function. The normality of residuals was assessed using the Shapiro-Wilk test and visual inspection of Q-Q plots UNIVARIATE procedure in SAS. The INFLUENCE option was used to analyze the residual variance and observations with an internally studentized residual ≥ 3 or ≤ −3 were treated as an outlier and removed from the dataset. The Satterthwaite method was used to test the denominator degrees of freedom for the fixed effects. The model statement for initial and final BW, ADG (excludes animals that died during the experiment; “deads out”), and all the blood analyses included the fixed effect of treatment, day (or period of days depending on the response variable), and treatment × day interaction. Pen(treatment) and heifer(pen) were used as random variables. Since no effect of treatment × run was detected for the main variables analyzed herein (e.g. DMI, WI, ADG, and health), run and load(run) were also included as random variables, since the objective here is not to evaluate the main effect of run (time of the year) on the response variables. The specified term for repeated statements was time, with heifer (pen × treatment) as the subject. The model statement for DMI, WI, and G: F included the fixed effect of treatment, day (or period of days depending on the response variable), and treatment × day interaction. The Pen(treatment), run, and load(run) were included as random variables. The specified term for repeated statements was day and Pen(treatment) was the subject. The covariance structure used was compound symmetry, which provided the best fit based on the smallest Alkaike information criterion. All results were reported as least square means using the LSMEANS option of SAS. When a treatment × time interaction was detected, the SLICE option of SAS was used to compare the treatments within each time point. Dunnett’s 2-tailed test for comparison with the control group was used. When a main effect of treatment effect was detected, Dunnett’s test was used to compare each treatment mean with the control mean. When an effect of day was detected, the means were averaged by day and compared using Tukey-Kramer test. Differences were considered significant when *P *≤ 0.05 and tendencies were determined if *P *> 0.05 and *P *≤ 0.10.

## Results

**Figure 1:**
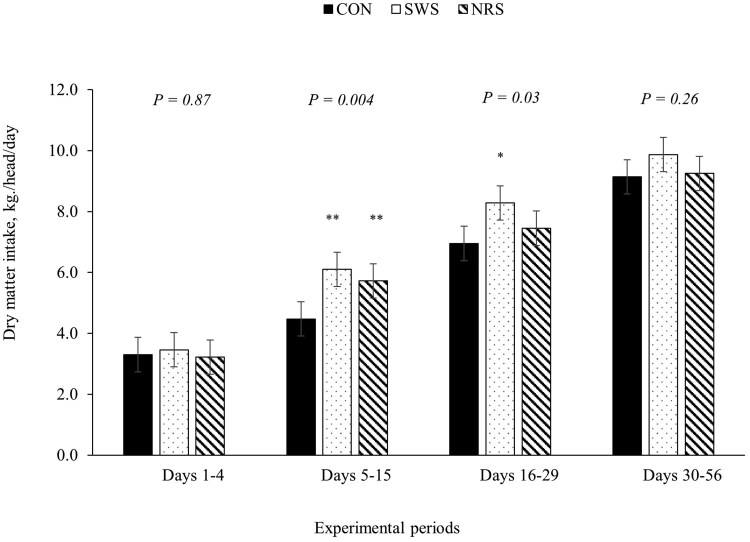
Dry matter intake of beef heifers during a 56-d receiving period provided with the following treatments during 4 d after the feedlot arrival: Control (CON): water was provided through standard in-pen automatic waterer only (CattleMaster 480; Richie Inc., Conrad, IA); Supplemental water source (SWS): CON plus water provided with one additional 416-L stock tank/pen (KMT 100; Tuff Stuff, Terra Bella, CA); Nutrient repletion solution (NRS): provided with one stock tank/pen (KMT 100; Tuff Stuff) as the only source of water. Treatments were provided from days 1 to 4, after which supplemental tanks were removed. From days 5 to 56, all heifers had only access to the standard in-pen automatic waterer (CattleMaster 480; Richie Inc.). Error bars represent the SEM. A treatment × experimental day interaction was detected for DMI (*P *= 0.03). Within each experimental period, means with symbols differ from the control treatment with Dunnett’s post hoc test (*P < 0.05; **P < 0.01).

**Figure 2:**
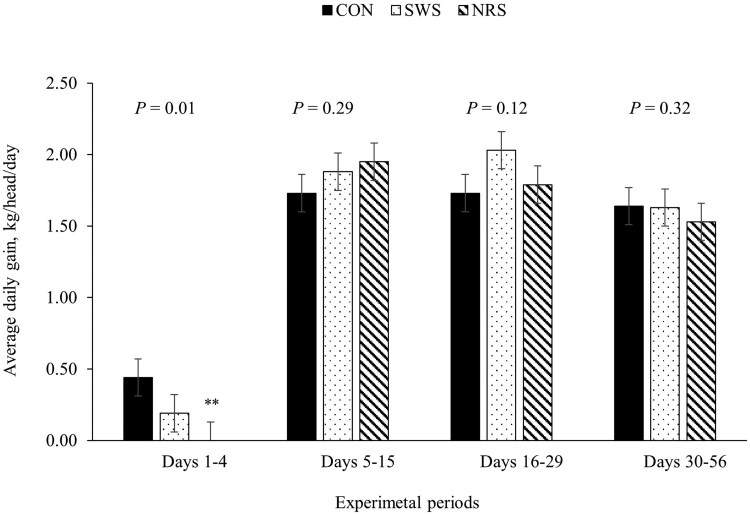
Average daily gain of beef heifers during a 56-d receiving period provided with the following treatments during 4 d after the feedlot arrival: Control (CON): water was provided through standard in-pen automatic waterer only (CattleMaster 480; Richie Inc., Conrad, IA); Supplemental water source (SWS): CON plus water provided with one additional 416-L stock tank/pen (KMT 100; Tuff Stuff, Terra Bella, CA); Nutrient repletion solution (NRS): provided with one stock tank/pen (KMT 100; Tuff Stuff) as the only source of water. Treatments were provided from days 1 to 4, after which supplemental tanks were removed. From days 5 to 56, all heifers had only access to the standard in-pen automatic waterer (CattleMaster 480; Richie Inc.). Error bars represent the SEM. A treatment × experimental day interaction was detected for ADG (*P *= 0.02). Within each experimental period, means with symbols differ from the control treatment with Dunnett’s post hoc test (**P < 0.01).

**Figure 3:**
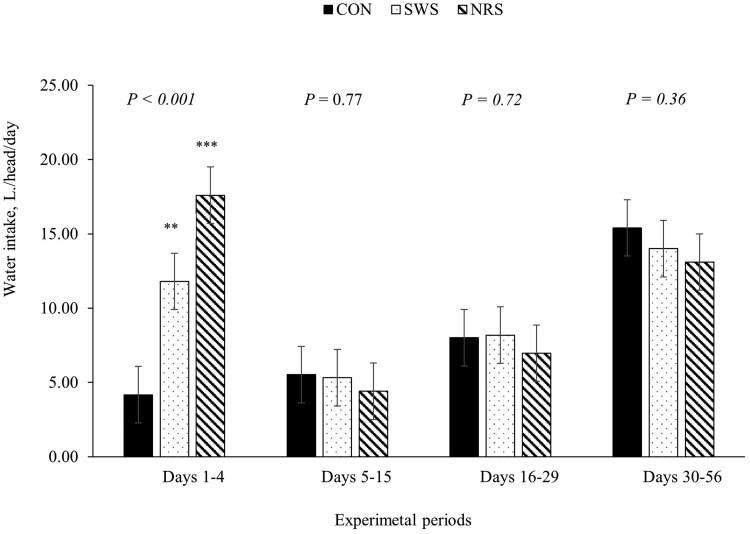
Water intake of beef heifers during a 56-d receiving period provided with the following treatments during 4 d after the feedlot arrival: Control (CON): water was provided through standard in-pen automatic waterer only (CattleMaster 480; Richie Inc., Conrad, IA); Supplemental water source (SWS): CON plus water provided with one additional 416-L stock tank/pen (KMT 100; Tuff Stuff, Terra Bella, CA); Nutrient repletion solution (NRS): provided with one stock tank/pen (KMT 100; Tuff Stuff) as the only source of water. Treatments were provided from days 1 to 4, after which supplemental tanks were removed. From days 5 to 56, all heifers had only access to the standard in-pen automatic waterer (CattleMaster 480; Richie Inc.). Error bars represent the SEM. A treatment × experimental day interaction was detected for WI (*P *< 0.001). Within each experimental period, means with symbols differ from the control treatment with Dunnett’s post hoc test (**P < 0.001).

**Figure 4:**
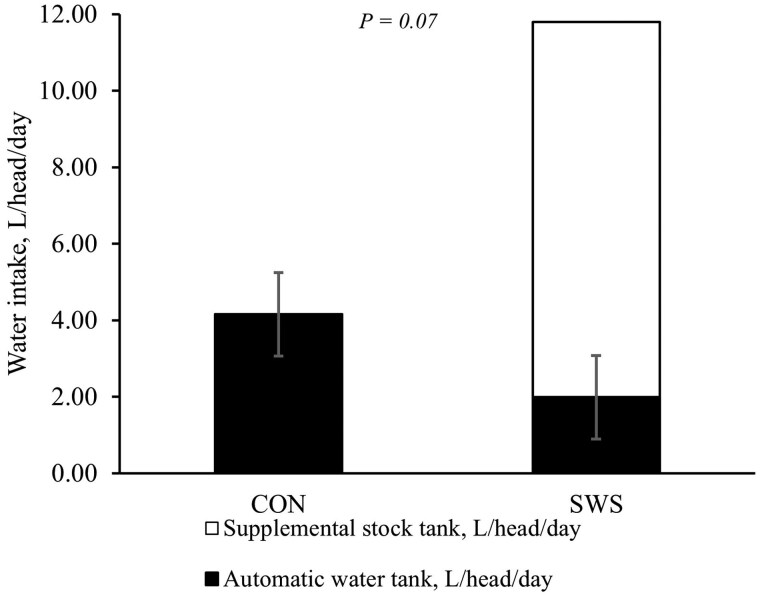
Water intake during 4 d after the feedlot arrival. Heifers were provided with the following treatments during 4 d after the feedlot arrival: Control (CON): water was provided through standard in-pen automatic waterer only (CattleMaster 480; Richie Inc., Conrad, IA); Supplemental water source (SWS): CON plus water provided with one additional 416-L stock tank/pen (KMT 100; Tuff Stuff, Terra Bella, CA); Nutrient repletion solution (NRS): provided with one stock tank/pen (KMT 100; Tuff Stuff) as the only source of water. Treatments were provided from days 1 to 4, after which supplemental tanks were removed. From days 5 to 56, all heifers had only access to the standard in-pen automatic waterer (CattleMaster 480; Richie Inc.). Error bars represent the SEM. A tendency (*P *= 0.07; SEM = 1.09) was observed in water intake from the automatic waterer (black bar) during the 4 d after feedlot arrival.

**Figure 5:**
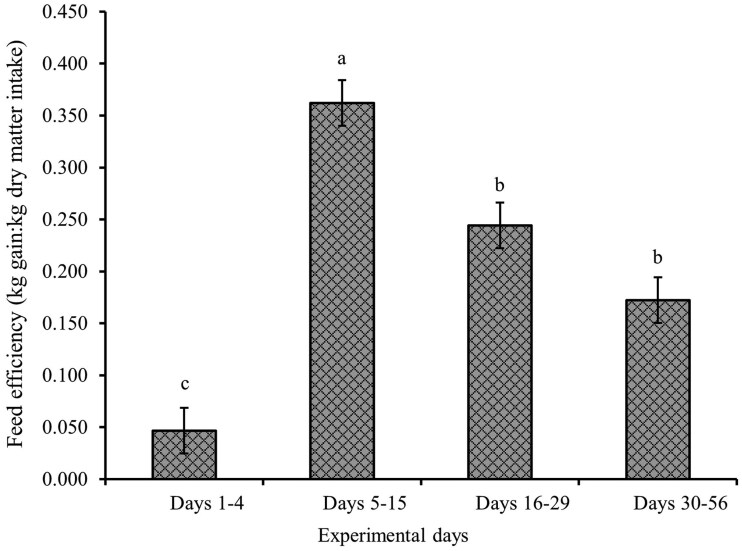
Feed efficiency (G: F) of beef heifers during a 56-d receiving period provided with the following treatments during 4 d after the feedlot arrival: Control (CON): water was provided through standard in-pen automatic waterer only (CattleMaster 480; Richie Inc., Conrad, IA); Supplemental water source (SWS): CON plus water provided with one additional 416-L stock tank/pen (KMT 100; Tuff Stuff, Terra Bella, CA); Nutrient repletion solution (NRS): provided with one stock tank/pen (KMT 100; Tuff Stuff) as the only source of water Treatments were provided from days 1 to 4, after which supplemental tanks were removed. From days 5 to 56, all heifers had only access to the standard in-pen automatic waterer (CattleMaster 480; Richie Inc.). Error bars represent the SEM. An effect of experimental day was detected for G: F (*P *< 0.001). Means with unlike superscripts differ with Tukey-Kramer post-hoc test (*P *≤ 0.05).

A treatment × day interaction was observed for average BW, ADG, and water intake (*P *≤ 0.05; Table 5). Providing SWS and NRS increased DMI compared to CON during days 5 to 15 (*P *< 0.001; Figure 1), and the increase in DMI persisted for SWS compared to CON between days 16 and 29 (*P *< 0.01; Figure 1). The ADG was lower for NRS compared to CON during days 1 to 4 (*P *< 0.01; Figure 2), and water intake was greater for SWS and NRS compared to CON between days 1 and 4 (*P *< 0.001; Figure 3). The increase in water intake observed for SWS compared to CON during days 1 to 4 was mainly due to the increase in water consumption from the supplemental water tank and a tendency to decrease water intake from the automatic water tank (*P *= 0.07; Figure 4). An effect of experimental day was observed for feed efficiency (*P *< 0.001; Table 5), which was lower during days 1 to 4, greatest during days 5 to 15, and intermediate from days 16 to 56 (*P *< 0.05; Figure 5).

**Table 5:** Growth performance and water intake of beef heifers during a 56-d receiving period provided with the experimental treatments during 4 d after the feedlot arrival.

Item	Treatments^1^			SEM^2^	*P*-values		
	CON	SWS	NRS		Treatment	Exp. Day	Treatment × Exp. Day
**Pens (heifers)**	6 (90)	6 (90)	6 (90)	–	–	–	–
**Body weight, kg**	277	278	276	6.81	0.73	<0.001	0.05
**Day 1**	232	231	232	6.91	0.99		
**Day 4**	234	232	231	6.90	0.77		
**Day 14**	252	253	252	6.91	0.94		
**Day 28**	278	283	276	6.92	0.18		
**Day 56**	324	326	320	6.92	0.24		
**Dry matter intake, kg/head/day**	7.30	8.32	7.75	0.48	0.06	<0.001	0.06
**Average daily gain, kg/head/day**	1.63	1.66	1.53	0.10	0.33	<0.001	0.02
**Feed efficiency^3^**	0.222	0.213	0.203	0.022	0.71	<0.001	0.32
**Water intake L/head/day**	10.9	10.7	10.1	1.73	0.20	<0.001	<0.001

1 Treatments were Control (CON): water was provided through standard in-pen automatic waterer only (CattleMaster 480; Richie Inc., Conrad, IA); Supplemental water source (SWS): CON plus water provided with one additional 416-L stock tank/pen (KMT 100; Tuff Stuff, Terra Bella, CA); Nutrient repletion solution (NRS): provided with one stock tank/pen (KMT 100; Tuff Stuff) as the only source of drinking solution. Treatments were provided from days 1 to 4, after which supplemental tanks were removed. From days 5 to 56, all heifers had only access to the standard in-pen automatic waterer (CattleMaster 480; Richie Inc.).

2 Highest pooled SEM was reported.

3 Gain-to-feed ratio (kg ADG/kg DMI).

The increase observed in water intake from days 1 to 4 (treatment phase) for SWS and NRS compared to control was mainly due to an increase in water intake during the morning and night periods (*P *< 0.001; Table 6).

**Table 6:** Water intake (WI) of beef heifers during the first 4 d after feedlot arrival.

	Treatments^1^				
Water intake (Days 1 to 4)	CON	SWS	NRS	SEM	*P*-value
**Pens (heifers)**	6 (90)	6 (90)	6 (90)	–	–
**WI, L/head/d**	4.16	11.8^**^	17.6^**^		<0.001
**WI, L/head (%)^2^**					
**0700 am to 1200 pm**	2.70 (65%)	4.63^*^ (34%)	7.08^**^ (40%)	0.571	<0.001
**1200 pm to 700 pm**	0.902 (21%)	2.61 (19%)	4.54^*^ (26%)	1.14	0.02
**0700 pm to 0700 am**	0.568 (14%)	6.43^**^ (47%)	6.01^**^ (34%)	0.862	<0.001

1 Treatments were control (**CON**; standard in-pen automatic water source, one waterer/pen), supplemental water source (**SWS**; CON + one additional stock tank/pen), and nutrient repletion solution (**NRS**; provided with one stock tank/pen). Treatments were provided from days 1 to 4, after which supplemental tanks were removed.

2 Values reported within the same column refer to the percentage of the total water intake.

Within the same line, means with symbols differ from the control treatment with Dunnett’s post hoc test (^*^P ≤ 0.01; ^**^P < 0.001).

**Figure 6:**
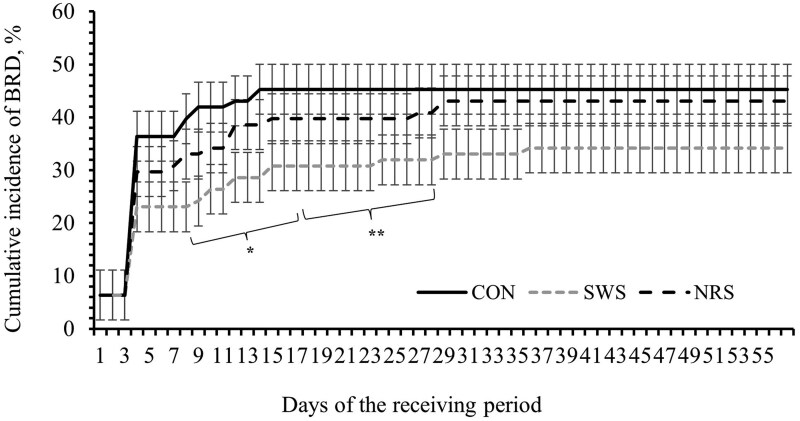
Cumulative incidence of Bovine Respiratory Disease (BRD) during a 56-d receiving period of heifers provided with the following treatments during 4 d after the feedlot arrival: Control (CON): water was provided through standard in-pen automatic waterer only (CattleMaster 480; Richie Inc., Conrad, IA); Supplemental water source (SWS): CON plus water provided with one additional 416-L stock tank/pen (KMT 100; Tuff Stuff, Terra Bella, CA); Nutrient repletion solution (NRS):provided with one stock tank/pen (KMT 100; Tuff Stuff) as the only source of water. Treatments were provided from days 1 to 4, after which supplemental tanks were removed. From days 5 to 56, all heifers had only access to the standard in-pen automatic waterer (CattleMaster 480; Richie Inc.). A treatment × experimental day interaction was detected (*P *= 0.02). Error bars represent the SEM. Means with symbols indicate a difference between CON and SWS with Dunnett’s post-hoc test (*P ≤ 0.10; **P ≤ 0.05).

No differences between treatments were observed for morbidity and mortality (*P *≥ 0.28; Table 7), although the 11% numerical decrease in the number of heifers that received one treatment against BRD in SWS compared to CON. The cumulative incidence of BRD was lower for SWS compared to CON during days 9 to 27 (*P *≤ 0.10; Figure 6).

**Table 7:** Morbidity and mortality of beef heifers during a 56-d receiving period provided with the experimental treatments during 4 d after the feedlot arrival.

	Treatments^1^				
Item	CON	SWS	NRS	SEM^2^	*P*-value
**Cattle treated for respiratory diseases, %**					
**First treatment**	43.2	32.0	40.9	22.4	0.28
**Second treatment**	13.9	11.7	16.1	8.29	0.64
**Third treatment**	2.80	6.11	2.78	3.16	0.49
**Mortality, %**	6.67	11.1	5.56	4.09	0.33
**Fatality rate, %^3^**	29.9	30.4	13.0	12.3	0.53

1 Treatments were Control (CON): water was provided through standard in-pen automatic waterer only (CattleMaster 480; Richie Inc., Conrad, IA); Supplemental water source (SWS): CON plus water provided with one additional 416-L stock tank/pen (KMT 100; Tuff Stuff, Terra Bella, CA); Nutrient repletion solution (NRS): provided with one stock tank/pen (KMT 100; Tuff Stuff) as the only source of water. Treatments were provided from days 1 to 4, after which supplemental tanks were removed. From days 5 to 56, all heifers had only access to the standard in-pen automatic waterer (CattleMaster 480; Richie Inc.).

2 Highest pooled SEM was reported.

3 Calculated as the number of deaths/number of treated calves in each pen.

**Figure 7:**
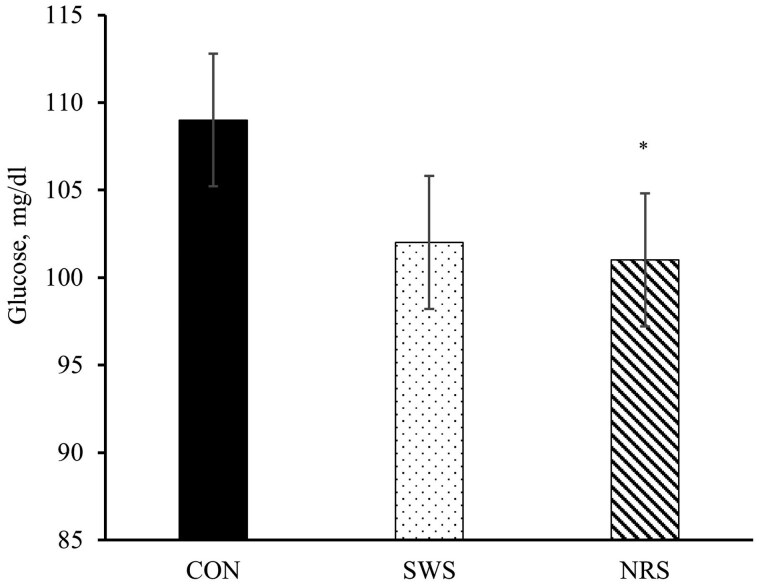
Plasma glucose concentrations of beef heifers during a 56-d receiving period provided with the following treatments during 4 d after the feedlot arrival: Control (CON): water was provided through standard in-pen automatic waterer only (CattleMaster 480; Richie Inc., Conrad, IA); Supplemental water source (SWS): CON plus water provided with one additional 416-L stock tank/pen (KMT 100; Tuff Stuff, Terra Bella, CA); Nutrient repletion solution (NRS): provided with one stock tank/pen (KMT 100; Tuff Stuff) as the only source of water. Treatments were provided from days 1 to 4, after which supplemental tanks were removed. From days 5 to 56, all heifers had only access to the standard in-pen automatic waterer (CattleMaster 480; Richie Inc.). Error bars represent the SEM. Blood samples were collected on days 1, 4, 14, 28, and 56 via jugular venipuncture from a cohort of 5 heifers/pen, based on the average initial body weight of each pen. Means with symbols indicate a difference between CON and NRS with Dunnett’s post-hoc test (*P = 0.09).

**Figure 8:**
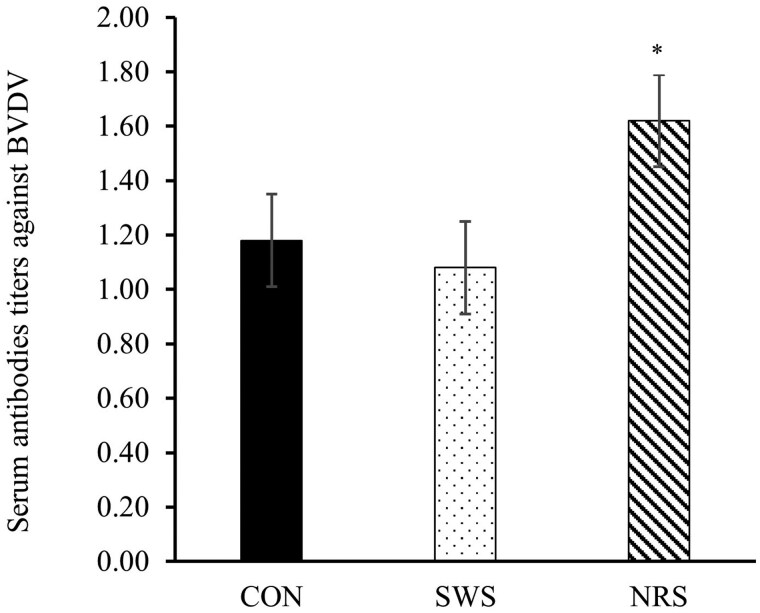
Serum antibodies titers against Bovine Viral Diarrhea Virus (BVDV) of beef heifers during a 56-d receiving period provided with the following treatments during 4 d after the feedlot arrival: Control (CON): water was provided through standard in-pen automatic waterer only (CattleMaster 480; Richie Inc., Conrad, IA); Supplemental water source (SWS): CON plus water provided with one additional 416-L stock tank/pen (KMT 100; Tuff Stuff, Terra Bella, CA); Nutrient repletion solution (NRS): provided with one stock tank/pen (KMT 100; Tuff Stuff) as the only source of water. Treatments were provided from days 1 to 4, after which supplemental tanks were removed. From days 5 to 56, all heifers had only access to the standard in-pen automatic waterer (CattleMaster 480; Richie Inc.). Error bars represent the SEM. Blood samples were collected on days 1, 4, 14, 28, and 56 via jugular venipuncture from a cohort of 5 heifers/pen, based on the average initial body weight of each pen. Means with symbols indicate a difference between CON and NRS with Dunnett’s post hoc test (*P = 0.02).

No treatment or treatment × day interactions were observed for any of the plasma hormones and metabolites evaluated (*P *≥ 0.11; Table 8), except for plasma glucose which tended to be lower for NRS compared to CON (*P *= 0.10; Figure 7), and serum antibodies titers against BVDV, which was greater for NRS compared to CON (*P *= 0.02; Figure 8).

**Table 8:** Physiological and humoral responses of beef heifers during a 56-d receiving period provided with the experimental treatments during 4 d after the feedlot arrival.

	Treatments^1^			SEM^2^	*P*-values		
Item^3^	CON	SWS	NRS		Treatment	Day	Treatment × Day
**Plasma hormone and metabolites**							
**Cortisol, ng/mL**	3.55	3.26	3.59	0.354	0.60	<0.001	0.99
**Haptoglobin, mg/mL**	0.446	0.460	0.460	0.083	0.96	<0.001	0.85
**Tumor necrosis factor-α, mg/mL**	4.91	4.49	4.04	1.12	0.78	0.14	0.35
**Nonesterified fatty acids, µEq/L**	0.487	0.527	0.503	0.124	0.72	<0.001	0.98
**β-hydroxybutyrate, mg/mL**	1.75	1.70	1.76	0.300	0.93	<0.001	0.12
**Lactate, mmol/L**	4.82	4.42	4.49	0.327	0.50	<0.001	0.93
**Gamma-glutamyl transferase, U/L**	15.0	13.2	14.6	0.843	0.31	<0.001	0.69
**Urea nitrogen, mg/dL**	13.1	12.3	12.8	2.72	0.57	<0.001	0.35
**Bicarbonate, mmol/L**	17.0	17.9	17.6	0.887	0.73	0.12	0.11
**Serum antibodies against respiratory viruses^4^**							
**Parainfluenza-3 virus**	59.6	70.8	72.1	26.1	0.60	0.01	0.65
**Bovine respiratory syncytial virus**	76.2	91.0	88.8	29.9	0.54	<0.001	0.85

1 Treatments were Control (CON): water was provided through standard in-pen automatic waterer only (CattleMaster 480; Richie Inc., Conrad, IA); Supplemental water source (SWS): CON plus water provided with one additional 416-L stock tank/pen (KMT 100; Tuff Stuff, Terra Bella, CA); Nutrient repletion solution (NRS): provided with one stock tank/pen (KMT 100; Tuff Stuff) as the only source of water solution. Treatments were provided from days 1 to 4, after which supplemental tanks were removed. From days 5 to 56, all heifers had only access to the standard in-pen automatic waterer (CattleMaster 480; Richie Inc.).

2 Highest pooled SEM was reported.

3 Blood samples were collected via jugular venipuncture on days 1, 4, 14, 28, and 56 from a cohort of 5 heifers/pen, based on the average initial body weight of each pen.

4 Only serum samples from heifers not treated for bovine respiratory disease during the experiment were analyzed.

**Figure 9:**
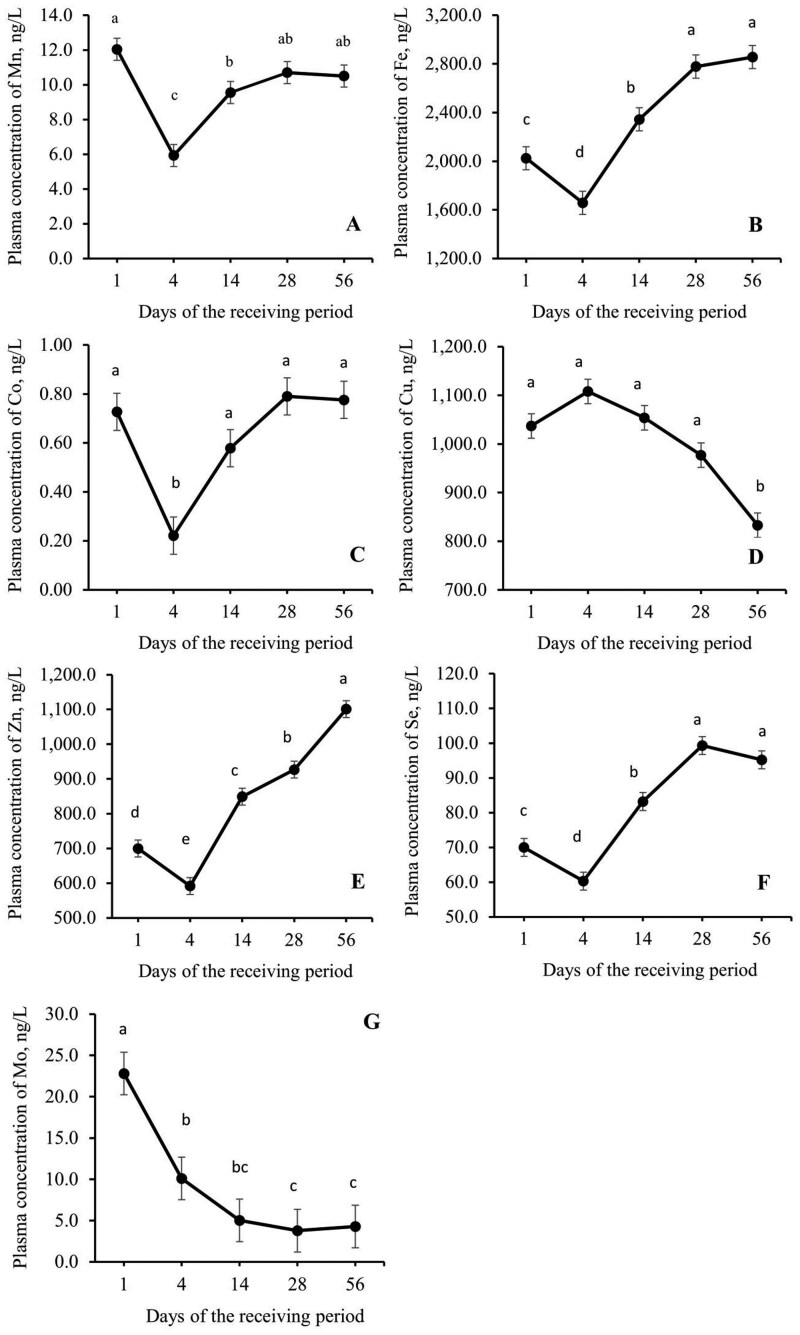
Plasma concentration (ng/L) of manganese (A), iron (B), cobalt (C), cooper (D), zinc (E), selenium (F), and molybdenum (G) of beef heifers during a 56-d receiving period provided with the experimental treatments during 4 d after the feedlot arrival. Error bars represent the SEM. Blood samples were collected on days 1, 4, 14, 28, and 56 via jugular venipuncture from a cohort of 5 heifers/pen, based on the average initial body weight of each pen. An effect of sampling day was observed (*P *< 0.001). Means with unlike superscripts differ with Tukey-Kramer post-hoc test (*P *≤ 0.05).

No treatment or treatment × day interactions (*P *≥ 0.13; Table 9) were observed for the complete blood count or plasma trace mineral concentrations (*P *≥ 0.11; Table 10), except for a tendency of treatment × day interaction for red blood cells which tended to be lower for SWS compared to CON on day 56 (*P *= 0.07; Table 9). A sampling day effect was observed for complete blood cell count and plasma trace mineral concentration (*P *≤ 0.05; Tables 9 to 11). Plasma concentration of several trace minerals (e.g. Mn, Fe, Co, Zn, and Se) decreased (*P *≤ 0.05) after the treatment phase, from day 1 to day 4, and then increased (*P *≤ 0.05) over the following days of the receiving period (Figure 9). Plasma concentration of Cu was lower at the end of the receiving period on day 56 (Figure 9D), and Mo was lower on days 28 and 56 (Figure 9G).

**Table 9:** Complete blood count of beef heifers during a 56-d receiving period provided with the experimental treatments during 4 d after the feedlot arrival.

	Treatments^1^			SEM^2^	*P*-values		
Item^3^	CON	SWS	NRS		Treatment	Day	Treatment × Day
**White blood cells, ng/L**	10.2	11.1	10.7	0.715	0.36	<0.001	0.12
**Red blood cells, ng/L**	10.0	9.66	9.88	0.292	0.54	<0.001	0.07
**Day 1**	11.0	10.6	10.8	0.377	0.56		
**Day 4**	9.90	9.70	9.58	0.377	0.69		
**Day 14**	9.60	9.37	9.41	0.377	0.81		
**Day 28**	9.60	9.24	9.35	0.377	0.63		
**Day 56**	10.1	9.38	10.3	0.377	0.07		
**Hemoglobin, g/dL**	11.4	10.8	11.0	0.277	0.24	<0.001	0.31
**Hematocrit, %**	40.2	38.1	38.8	0.805	0.20	<0.001	0.83
**Mean corpuscular volume, fl^4^**	40.4	39.9	39.7	0.680	0.76	<0.001	0.38
**Mean corpuscular hemoglobin, g/dL^4^**	11.5	11.3	11.2	0.171	0.50	<0.001	0.62
**Mean corpuscular hemoglobin concentration, g/dL^4^**	28.4	28.3	28.2	0.215	0.64	<0.001	0.15
**Platelet, ×10^3^/μL**	385	383	379	20.8	0.97	<0.001	0.73
**Lymphocyte, ×10^3^/μL**	6.43	7.00	6.57	0.795	0.49	<0.001	0.70
**Monocyte, ×10^3^/μL**	0.494	0.578	0.614	0.142	0.32	<0.001	0.97
**Neutrophil, ×10^3^/μL**	3.17	3.36	3.17	0.273	0.84	0.05	0.13
**Eosinophil, ×10^3^/μL**	0.156	0.174	0.185	0.014	0.36	<0.001	0.19
**Basophils, ×10^3^/μL**	0.066	0.074	0.073	0.005	0.51	<0.001	0.57
**Netrophil:lymphocyte ratio**	0.569	0.554	0.546	0.099	0.93	0.71	0.55

1 Treatments were Control (CON): water was provided through standard in-pen automatic waterer only (CattleMaster 480; Richie Inc., Conrad, IA); Supplemental water source (SWS): CON plus water provided with one additional 416-L stock tank/pen (KMT 100; Tuff Stuff, Terra Bella, CA); Nutrient repletion solution (NRS): provided with one stock tank/pen (KMT 100; Tuff Stuff) as the only source of water. Treatments were provided from days 1 to 4, after which supplemental tanks were removed. From days 5 to 56, all heifers had only access to the standard in-pen automatic waterer (CattleMaster 480; Richie Inc.).

2 Highest pooled SEM was reported.

3 Blood samples were collected on days 1, 4, 14, 28, and 56 via jugular venipuncture from a cohort of 5 heifers/pen, based on the average initial body weight of each pen.

4 MCV = Mean corpuscular volume; Mean corpuscular hemoglobin; MCHC = Mean corpuscular hemoglobin concentration.

**Table 10:** Plasma mineral concentrations of beef heifers during a 56-d receiving period provided with the experimental treatments during 4 days after the feedlot arrival.

	Treatments^1^			SEM^2^	*P*-values		
Item^3^	CON	SWS	NRS		Treatment	Day	Treatment × Day
**Iron, ng/L**	2,354	2,260	2,381	255	0.40	<0.001	0.18
**Copper, ng/L**	1,036	974	996	128	0.34	<0.001	0.39
**Zinc, ng/L**	842	820	839	44.2	0.80	<0.001	0.11
**Cobalt, ng/L**	0.650	0.656	0.549	0.358	0.34	<0.001	0.57
**Manganese, ng/L**	10.3	9.54	9.39	3.02	0.21	<0.001	0.35
**Selenium, ng/L**	81.4	81.9	81.5	3.62	0.98	<0.001	0.25
**Molybdenum, ng/L**	8.45	10.7	8.49	4.60	0.49	<0.001	0.92

1 Treatments were Control (CON): water was provided through standard in-pen automatic waterer only (CattleMaster 480; Richie Inc., Conrad, IA); Supplemental water source (SWS): CON plus water provided with one additional 416-L stock tank/pen (KMT 100; Tuff Stuff, Terra Bella, CA); Nutrient repletion solution (NRS): provided with one stock tank/pen (KMT 100; Tuff Stuff) as the only source of water. Treatments were provided from days 1 to 4, after which supplemental tanks were removed. From days 5 to 56, all heifers had only access to the standard in-pen automatic waterer (CattleMaster 480; Richie Inc.).

2 Highest pooled SEM was reported.

3 Blood samples were collected on days 1, 4, 14, 28, and 56 via jugular venipuncture from a cohort of 5 heifers/pen, based on the average initial body weight of each pen.

**Table 11:** Effect of day on complete blood count of beef heifers during a 56-d receiving period.

Item	Days of the receiving period^1^					SEM^2^	*P*-value
	1	4	14	28	56		
**White blood cells, ng/L**	11.7^a^	9.83^c^	9.60^c^	11.9^a^	10.3^b^	0.395	<0.001
**Hemoglobin, g/dL**	11.8^a^	10.5^c^	10.1^d^	10.9^b^	12.0^a^	0.119	<0.001
**Hematocrit, %**	41.7^a^	37.9^a^	36.3^b^	37.8^b^	41.6^a^	0.473	<0.001
**Mean corpuscular volume, fl^4^**	38.9^c^	39.4^c^	39.0^c^	40.8^b^	42.1^a^	0.25	<0.001
**Mean corpuscular hemoglobin, g/dL^4^**	11.0^c^	10.9^c^	10.8^c^	11.8^b^	12.0^a^	0.083	<0.001
**Mean corpuscular hemoglobin concentration, g/dL^4^**	28.2^b^	27.9^c^	27.6^c^	28.9^a^	28.9^a^	0.201	<0.001
**Platelet, ×10^3^/μL**	379^a^	323^b^	396^a^	408^a^	407^a^	19.8	<0.001
**Lymphocyte, ×10^3^/μL**	7.44^a^	6.13^b^	6.09^b^	7.32^a^	6.37^b^	0.313	<0.001
**Monocyte, ×10^3^/μL**	0.767^a^	0.575^b^	0.467^b,c^	0.656^a^	0.346^c^	0.065	<0.001
**Neutrophil, ×10^3^/μL**	3.39^a,b^	3.06^a,b^	2.92^b^	3.52^a^	3.28^a,b^	0.223	<0.001
**Eosinophil, ×10^3^/μL**	0.200^a^	0.128^b^	0.142^b^	0.204^a^	0.184^a,b^	0.018	<0.001
**Basophils, ×10^3^/μL**	0.077^a,b^	0.044^b^	0.061^b^	0.095^a^	0.079^a^	0.006	<0.001

1 Treatments were Control (CON): water was provided through standard in-pen automatic waterer only (CattleMaster 480; Richie Inc., Conrad, IA); Supplemental water source (SWS): CON plus water provided with one additional 416-L stock tank/pen (KMT 100; Tuff Stuff, Terra Bella, CA); Nutrient repletion solution (NRS): provided with one stock tank/pen (KMT 100; Tuff Stuff) as the only source of water. Treatments were provided from days 1 to 4, after which supplemental tanks were removed. From days 5 to 56, all heifers had only access to the standard in-pen automatic waterer (CattleMaster 480; Richie Inc.). Blood samples were collected on days 1, 4, 14, 28, and 56 of the experiment.

2 Highest pooled SEM was reported.

3 Blood samples were collected on days 1, 4, 14, 28, and 56 via jugular venipuncture from a cohort of 5 heifers/pen, based on the average initial body weight of each pen.

a,b,c,d Within a row, means with unlike superscripts differ with Tukey-Kramer post-hoc test (*P *≤ 0.05).

Pearson correlation coefficient showed a positive correlation between DMI and water intake (0.37675; *P *< 0.001) and a negative correlation between DMI and THI (−0.11746; *P *= 0.002; data not shown).

## Discussion

Little information is published concerning WI of newly received feedlot cattle. In the current study, overall WI was 10.9, 10.7, and 10.1 L/head/day for CON, SWS, and NRS, respectively, during the 56-d receiving experiment period, with the greatest WI occurring during the first 4 d following feedlot arrival when treatments were offered. Winchester and Morris (1956) were the first to develop a model to calculate total WI for feedlot cattle using DMI, predicted WI, and average ambient temperature. Using the Winchester and Morris (1956) model, we estimated total WI for the current study would average 19.22 L/head/day with ambient temperature averaging 14.4 °C. Hicks et al. (1988) developed an equation to estimate WI using a model that incorporates average daily maximum ambient temperature, DMI, precipitation, and dietary salt content. Using Hicks et al. (1988) model, we estimated total WI for the current study would average 9.31/head/day with an average daily maximum ambient temperature of 15.5 °C. The WI reported by Winchester and Morris (1956) was greater than those observed in the current study, whereas the WI estimated by Hicks et al. (1988) was similar to the actual WI recorded in the current study. Winchester and Morris (1956) model is more accurate for forecasting WI in large herds than in small herds, and DMI more accurately predicts WI in extreme environmental conditions than in moderate environmental conditions. While WI and DMI were measured at the pen level, with WI data derived from water flow measurements, the Pearson correlation coefficient between DMI and WI observed in the current study aligns with findings by Ahlberg et al. (2019), who utilized individual data collected through the Insentec system (Hokofarm Group, The Netherlands).

The increase in WI during the first 4 d following feedlot arrival for NRS (9.0 cm water trough space per head) and SWS (17.6 cm water trough space per head) compared to CON (8.7 cm water trough space per head) may be attributed to the supplemental stock tanks offered to NRS as the only water source and to SWS as a supplemental water source when compared to CON with the automatic water as the only water source. Water intakes decreased after the removal of the supplemental stock tanks on day 4, and from days 4 to 56, all heifers had only access to the standard in-pen automatic waterers. The trough type may have had an impact on drinking behavior and overall WI, which could explain the higher intake from SWS and NRS. Coimbra et al. (2010) observed greater water intake from a round polyvinyl chloride water tank with a surface area of 1.13 m^2^ than from a rectangular concrete trough with a surface area of 0.75 m^2^ when heifers had access to both troughs. In the current study, heifers favored the larger supplemental stock tanks with a surface area of 1.23 m^2^ over the smaller automatic waterers with a surface area of 0.44 m^2^, which was consistent with Coimbra et al. (2010). Heifers may have preferred the supplemental stock tanks to the automatic waterers due to a variety of factors, but the greater surface area, which allowed more cattle to congregate around the tank, was probably the main reason for the increased WI. Pinheiro Machado Filho et al. (2004) and Teixeira et al. (2006) observed drinking behaviors of dairy cows and determined that cattle prefer larger troughs with more water surface and depth when presented with options. In comparison to a smaller water source, cattle have increased WI, visit more frequently, and drink for longer periods of time (Coimbra et al. 2010). According to Seymour et al. (2024), beef cattle provided with an electrolyte solution during the receiving period consumed 47% more fluid compared to the control group. The electrolyte composition may drive initial drinking through osmotic stimulation in the current experiment. According to Unny et al. (2023), electrolytes, particularly sodium and chloride, are key determinants of extracellular fluid osmolarity. When osmolarity rises, hypothalamic osmoreceptors detect the shift and activate mechanisms that provoke thirst and promote drinking behavior, increasing water intake and per or frequency of drinking as observed in the current study. There is very little research on beef cattle in terms of water supply compared to dairy cows, and more research is needed to determine the impact of water sources on feedlot cattle WI.

The lack of immediate effects of treatments on DMI during the first 4 d can likely be attributed to transient anorexia resulting from shipping stress and its physiological consequences. Transport and handling activate the hypothalamic–pituitary–adrenal (HPA) axis, leading to elevated cortisol and the release of pro-inflammatory cytokines such as TNF-α, IL-6, and IL-8, which suppress appetite by acting on hypothalamic centers that regulate feeding behavior (Gouvêa et al. 2022). This stress-induced anorexia, combined with dehydration and ruminal disruption, likely contributed to increased water intake, particularly in treatments involving electrolyte supplementation or additional trough access, while ADG remained suppressed as cattle prioritized rehydration and immune stabilization over nutrient intake and tissue accretion.

Water is the most critical nutrient required for many metabolic reactions in the body (National Academies of Sciences, Engineering, and Medicine (U.S.). Committee on Nutrient Requirements of Beef Cattle 2016). Because dehydration weakens the innate immune response and reduces resistance to pathogens (Ackermann et al. 2010), significant loss of water and key nutrients from the body can put the animal at increased risk for BRD (Carroll and Forsberg 2007). Normal bovine hematocrit levels should be between 25% and 33% (Cornell University College of Veterinary Medicine 2019). In this study, dehydration was indicated by elevated hematocrit percentages (42.6%, 40.9%, and 41.7% for CON, SWS, and NRS, respectively) in heifers on day 1, which is consistent with Richeson et al. (2013) and Oosthuysen et al. (2017), who observed elevated hematocrit percentages (36.9% and 38.5%, respectively) in newly received beef calves arriving at the feedlot. Reduced hematocrit percentages measured on day 4 (39.1%, 37.7%, and 37.2% for CON, SWS, and NRS, respectively) corresponded to what was hypothesized to reflect increased WI from days 1 to 4 for both SWS and NRS. Although hematocrit percentages decreased on day 4, they were still well above the normal range recommended by Cornell University College of Veterinary Medicine (2019). Pompek et al. (2024) evaluated the rehydration of neonatal dairy calves 3 to 7 d of age following transport. It was observed in that study that 90% of the calves had mild to moderate dehydration on arrival based on the skin tent test and blood biochemical indicators. Pompek et al. (2024) evaluated hematocrit levels to assess dehydration upon arrival; however, hematocrit levels were within the normal range, suggesting that hematocrit levels alone may not be the most reliable indicator of dehydration. This study did observe that 76.5% of the calves were hypoglycemic on arrival (glucose < 4.95 mmol/L). Based on hematocrit values alone, calves in the current experiment were still dehydrated after the 56-d receiving period. Maybe hematocrit alone is not the best predictor of the hydration status in receiving beef calves. This limitation likely reflects the influence of other factors such as stress, hemoconcentration, and metabolic adjustments during the receiving phase. Therefore, hematocrit should be interpreted in conjunction with additional indicators of hydration and health. Significant energy deficits brought on by extended fasting and stress during transportation are indicated by the high prevalence of hypoglycemia. Elevated hematocrit and hypoglycemia observed herein highlight the necessity of efficient rehydration and nutritional measures to reduce transport stress and enhance calf health. Although there were no main effects of treatment or treatment by day for plasma cortisol and haptoglobin concentrations, the day effects detected indicate that heifers were exposed to the physiological effects of stress as seen by the adrenocortical and acute-phase protein responses due to the marketing and transportation process (Lippolis et al. 2017). There was no effect of treatment or day detected for TNF-alpha. It’s possible the current study found no such effect for TNF-alpha due to sampling by day rather than by hour. Waggoner et al. (2009) reported TNF-alpha concentrations peaked 2 hours following bacterial lipopolysaccharide administration and concentrations returned to baseline by hour 6, implying that TNF-alpha has a short half-life. Due to the less time-intensive sampling method used in the current study, it’s feasible that an increase in TNF-alpha concentration was missed. During the acute phase response, TNF-alpha is released in response to specific receptors on target tissues to reduce fever, leukocytosis, increase glucocorticoid secretion, regulate blood coagulation, and cause changes in the concentration of acute phase proteins (APP) (Heinrich et al. 1990). Changes in blood plasma APP levels (e.g. Hp) occur at a significantly lower rate when compared to other biomarkers of the acute phase response (Godson et al. 1996). Based on the diet composition and DMI observed in the current experiment, nutrient requirements were exceeded, although a positive effect of NRS was observed in antibody titers against BVDV. Zinc fortification seems to be beneficial for lightweight receiving cattle. Providing 120 mg/kg DM of supplemental dietary zinc resulted in increased DMI and ADG of lightweight calves (initial BW = 297 kg) after an 18-h transit event (Heiderscheit and Hansen 2022). Trace minerals supplementation (ZN, Mn, Cu, and Co) also positively influenced the humoral responses of weaned calves at feedlot arrival (Smercheck et al. 2023), and the organic source of trace minerals (amino acid complex) increased ADG and G: F (Smercheck et al. 2023). The observed increase in BVDV antibody titers in calves receiving the NRS is likely attributable to the combined effects of its active components. Organic trace minerals such as zinc, copper, and manganese play critical roles in immune function, including lymphocyte proliferation, antioxidant defense, and antibody synthesis (Smercheck et al. 2023; Nair et al. 2025). Previous studies have demonstrated that supplementation with trace minerals during vaccination enhances both the magnitude and speed of antibody responses to BVDV. Palomares et al. (2016) reported that injectable trace minerals (Zn, Cu, Mn, Se) administered concurrently with respiratory vaccines improved antibody titers and leukocyte function. Similarly, Roberts et al. (2016) observed higher BVDV-specific antibody titers in feedlot calves supplemented with trace minerals at processing. The inclusion of energy substrates such as glycerol and propylene glycol in the NRS may have supported immune function by providing readily available energy during periods of stress and immune activation. Glycerol and propylene glycol are quickly fermented in the rumen into volatile fatty acids, notably propionate, which supports gluconeogenesis and provides essential energy for antibody production and immune activation (Trabue et al. 2007). This likely reflects increased glucose utilization for immune activation and tissue metabolism rather than impaired gluconeogenesis.

Although no treatment or treatment × day effects were reported for plasma glucose, NEFA, BHBA, GGT, BUN, and LACT, day effects were observed and indicated how the nutritional and metabolic status of heifers changed following their arrival at the feedlot. In beef feedlot cattle, glucose concentrations are linked to DMI, nutrient availability, and BW gain. Inadequate nutrient intake, elicited by insufficient energy balance and body fat reserves, is linked to NEFA and BHBA concentrations (Hess et al. 2005). During the 56-d receiving period, the day effect on blood metabolites reported herein accurately depicts DMI and improved utilization of dietary nutrients as perceived by the experimental design, resulting in increased BW, improved ADG, and feed efficiency. In agreement with the current study hypothesis, increased water intake after feedlot arrival increased DMI during days 5 to 15 (for SWS and NRS groups) and days 16 to 29 (for SWS group) of the experiment. A decrease in BRD incidence for the SWS group was also observed during this same period. However, in contrast with the hypothesis, no morbidity or mortality differences among treatments were observed despite the numerical increase in the number of calves supplied SWS that required a third antimicrobial treatment for BRD and the numerical increase in mortality compared with CON. Tomczak et al. (2019) found that calves drenched with water upon arrival had an increase in mortality despite the decrease in days to the first BRD treatment, implying that oral hydration therapy may have improved the calf’s immune response to infection, resulting in increased mortality due to earlier clinical BRD recognition and treatment. It is important to note that in the study by Tomczak et al. (2019), water was administered via drenching (i.e. forced-feeding), which differs from the current study where animals had free access to water and could drink at their own discretion. Still in Tomczak et al. (2019), there was an experimental period effect detected for PI3, BVDV, and BRSV, indicating that there was an improvement in acquired immunity over the duration of the 56-d receiving period. While communal water tanks are a standard feature in commercial feedlot operations, typically serving large groups of cattle (e.g. ∼100 to 200 animals per pen), they may also present a potential route for pathogen transmission, especially under conditions of stress or immunosuppression. According to Beauvais et al. (2018), feedlot pens with higher cattle-to-water ratios exhibited increased fecal shedding of *E. coli* O157: H7, indicating shared troughs can elevate pathogen load and risk. Given the scale and practicality of feedlot management, isolating water sources is not feasible, but this factor warrants consideration in interpreting health outcomes. The lack of published information regarding the effects of hydration status and WI on humoral immunity against BRD pathogens in receiving calves following feedlot arrival necessitates further investigation.

In summary, providing SWS or NRS during the initial 4 d after feedlot arrival to high-risk newly received beef calves can increase water and feed intake. Supplementing NRS enhanced immune response by increasing antibody titers against BVD, which can be beneficial to decrease the negative effects of BRD during the feedlot receiving period.

## Abbreviations:

ADF: acid detergent fiber
ADG: average daily gain
BHBA: b-hydroxybutyrate
BRD: bovine respiratory disease
BRSV: bovine respiratory syncytial virus
BUN: blood urea nitrogen
BVDV: bovine viral diarrhea virus
BW: body weight
CBC: complete blood count
CO_2_: carbon dioxide
CON: control
CP: crude protein
DART: depression anorexia respiration temperature
DM: dry matter
DMI: dry matter intake
FE: feed efficiency
G: F: gain to feed ratio
GGT: gamma-glutamyl transferase
HBG: hemoglobin
HCT: hematocrit
Hp: haptoglobin
HRP: horseradish peroxidase
K2 EDTA: dipotassium ethylenediaminetetraacetic acid
LACT: lactic acid
Mcal: mega calorie
NDF: neutral detergent fiber
NEFA: non-esterified fatty acid
NE_g_: net energy of gain
NE_m_: net energy of maintenance
NRS: nutrient repletion solution
PI3: parainfluenza-3 virus
QC: quality control
RBC: red blood cell
SWS: supplemental water source
TDN: total digestible nutrients
TNF-alpha: tumor necrosis factor alpha
WBC: white blood cell
WI: water intake
